# Salvage boron neutron capture therapy for recurrent intracranial malignant meningioma: Case report and literature review

**DOI:** 10.1097/MD.0000000000043743

**Published:** 2025-08-01

**Authors:** Li-Ching Lin, Wan-Hsuan Chow, Ya-Ju Wu, Yuan-Hung Wu, Wan-Lin Shen, Chin-Hong Chang, Sheng-Yow Ho

**Affiliations:** a Department of Radiation Oncology, Chi Mei Medical Center, Tainan, Taiwan; b Department of Pathology, Chi-Mei Medical Center, Tainan, Taiwan; c Division of Radiation Oncology, Department of Oncology, Taipei Veterans General Hospital, Taipei, Taiwan; d School of Medicine, College of Medicine, National Yang Ming Chiao Tung University, Taipei, Taiwan; e Department of Neurosurgery, Chi Mei Medical Center, Tainan, Taiwan.

**Keywords:** boron neutron capture therapy (BNCT), malignant meningioma, radiotherapy, targeted therapy

## Abstract

**Rationale:**

Meningiomas are the most common central nervous system tumors in adults, accounting for approximately one-third of the total. Although only 1% to 3% of meningiomas are malignant, these cases exhibit a high degree of aggression and frequent recurrence, resulting in a poor prognosis. There are no accepted treatment standards for salvage re-irradiation. Therefore, detailed case descriptions are essential for treatment guidance.

**Patient concerns:**

A 56-year-old female with malignant meningioma underwent curative resection and adjuvant radiotherapy (66 Gy in 33 fractions). However, the tumor recurred repeatedly despite curative and salvage therapies. The safety and efficacy of salvage re-irradiation may be limited by potential brain toxicity.

**Diagnoses:**

Recurrent malignant meningioma.

**Interventions:**

The meningioma recurred at the surgical dural bed 4 months after adjuvant radiotherapy, as evidenced by histological analysis following salvage craniotomy. Progressive meningioma was also evident, with invasion to the adjacent skull bone and scalp. Compassionate salvage boron neutron capture therapy (BNCT) was prescribed to eradicate both the recurrent meningioma and bone invasion. The 2 courses of BNCT were administered with mean tumor doses of 41.52 and 50.22 GyE.

**Outcomes:**

No clinically significant toxicity was observed following irradiation, and imaging showed near-total regression of the brain meningioma and the adjacent invasive skull bone and scalp tumor. Brain symptoms of headache and dizziness also abated after treatment.

**Lessons:**

A favorable tumor response with no cranial toxicity was achieved using targeted BNCT therapy. We review the literature on recurrent malignant meningioma and relevant BNCT salvage for further therapeutic guidance.

## 1. Introduction

Meningiomas are the most common central nervous system tumors in adults. The World Health Organization categorizes meningiomas into 3 histological grades: grade I (benign), grade II (atypical), and grade III (malignant or anaplastic). Most meningiomas are benign (80%–90%), while only 1% to 3% are malignant. However, these malignant cases are highly aggressive, prone to recurrence, and have relatively poor progress.^[1–3]^ Maximal safe resection followed by adjuvant radiotherapy is the treatment of choice for atypical and malignant meningioma because the efficacy of radiotherapy for improved local control of both benign and nonbenign meningiomas has been demonstrated by phase II and retrospective studies.^[4]^ Unfortunately, despite initial treatment success, most atypical and malignant meningiomas eventually relapse within the previously resected dural margin^[4,5]^ and require more aggressive salvage treatment.

The vast majority of recurrent meningiomas are confined to the previous tumor bed, so salvage resection and irradiation are potential treatment options. These re-irradiation options include brachytherapy, conventional fractionated radiation therapy, stereotactic radiosurgery, proton beam irradiation, and boron neutron capture therapy (BNCT).^[6–8]^ However, there are limited data on the safety and efficacy of salvage re-irradiation, and normal brain dose may be constrained by possible toxicity.

BNCT, reported as a targeted salvage radiotherapy, leverages the higher uptake of boron-containing drugs by malignant tumor cells compared to normal brain tissues. This allows for the delivery of a higher therapeutic dose to previously treated brain regions.^[8–12]^ Herein, we report a rare case of malignant meningioma with repeated cranial recurrence that was treated with salvage BNCT to address brain recurrence. Additionally, we review the literature on recurrent meningioma and explore the potential of targeted brain therapy as a salvage option.

## 2. Case presentation

**Figure 1.**
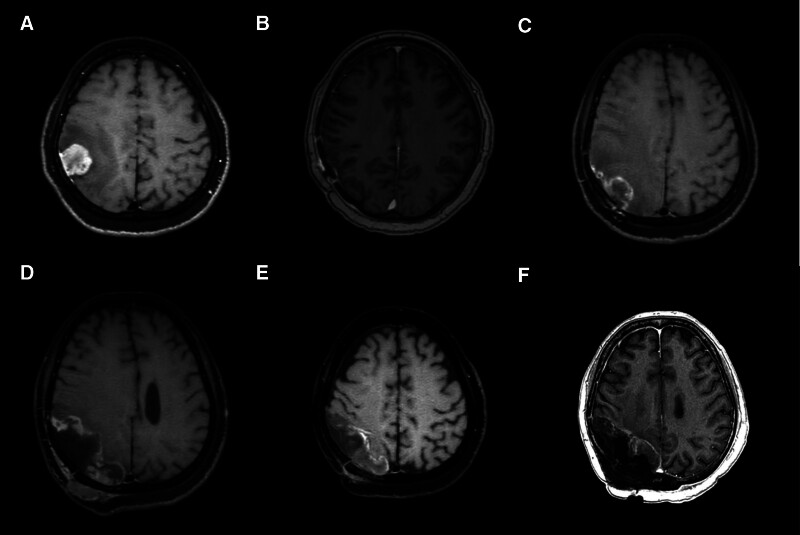
Preoperative magnetic resonance imaging (MRI) results showing primary and recurrent meningioma in the case patient. (A) T1-weighted image showing a homogeneously Gd-enhancing, well-defined meningioma in the right parietal region accompanied by substantial peritumoral edema. (B) Follow-up image after surgery showing no detectable residual tumor. (C) Recurrence at the previous surgical dural bed with significant perifocal edema. (D) Second recurrence showing downward extension to the parieto-occipital region and outward invasion to adjacent skull bone and scalp. (E, F) Follow-up image 3 and 10 months respectively after second boron neutron capture therapy showing nearly total regression of the recurrent meningioma and invasive skull bone and scalp tumor. Gd = gadolinium.

**Figure 2.**
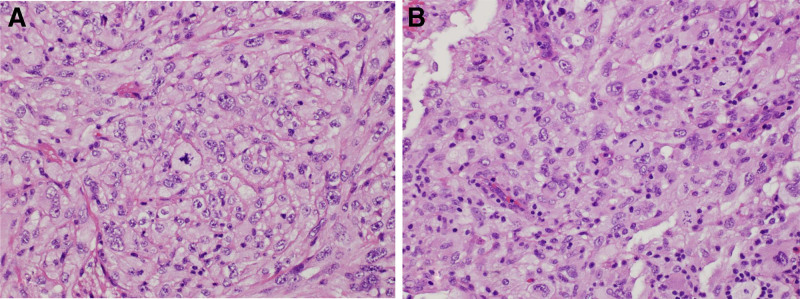
Histopathology of resected tumors. (A) Primary meningioma. (B) Pulmonary metastasis showing similar carcinoma- and melanoma-like cytomorphology with increased mitotic figures. Cytomorphology revealed by hematoxylin and eosin staining.

A 56-year-old female with a history of hypertension and dyslipidemia presented at the emergency department in April 2022 with acute-onset left hand clumsiness and numbness. Brain magnetic resonance imaging (MRI) detected a well-defined, gadolinium-enhanced mass attached to the dura in the right parietal region that was roughly 23 × 23 × 25mm^3^ in volume and accompanied by substantial peritumoral edema (Fig. 1A). Chest computed tomography (CT) excluded possible metastasis from lung cancer. Meningeal tumor was suspected and craniotomy was conducted for gross tumor excision in April 2022. Histological examination of the excised specimen confirmed meningeal tumor with atypical epithelioid, spindle meningothelial-like, and melanoma-like cytomorphology; evident nuclear pleomorphisms; elevated mitosis; and necrotic foci; and malignant meningioma was diagnosed based on these features (Fig. 2A). The patient received adjuvant radiotherapy to the surgical tumor bed (66 Gy in 33 fractions) from May to July 2022. The course of surgery and adjuvant radiotherapy was unremarkable, and follow-up imaging did not detect gross residual tumor (Fig. 1B).

However, the patient developed slurred speech and left limb weakness in November 2022, and a follow-up brain MRI revealed a growing, gadolinium-enhanced tumor about 36-mm in length at the previous surgical dural bed with substantial perifocal edema (Fig. 1C). Salvage craniotomy and removal of the tumor were performed again in December 2022, and the cytopathology was similar to that of the previous specimen, consistent with recurrent malignant meningioma. The patient began treatment with temozolomide and the antiangiogenic agent bevacizumab starting in January 2023.

Left side weakness recurred in March 2023, accompanied by scalp protrusion in the region of the previous craniotomy, and brain MRI revealed recurrent meningioma extending downward to the parieto-occipital region and possible invasion to the adjacent skull bone and scalp (Fig. 1D). Neither a second salvage craniotomy nor re-irradiation was recommended due to the potential for further brain damage, so the patient selected BNCT as compassionate salvage treatment. Initial 4-borono-2-^18^F-fluoro-phenylalanine positron emission tomography was conducted to ensure that the therapeutic agent (4-^10^B-borono-l-phenylalanine) selectively targeted the tumor over normal brain tissue.^[13]^ The first course of BNCT was conducted in April 2023 and involved a mean tumor dose of 41.52 GyE. No clinically substantial toxicity was noted after irradiation, and images acquired in September 2023 demonstrated near total regression of the brain meningioma as well as the adjacent invasive skull bone and scalp tumor. Headache and dizziness also abated after treatment. Unfortunately, the patient again presented with dizziness and left arm weakness in November 2023, and repeated brain MRI revealed mildly enhanced tumor progression in the right posterior parietal region. A second course of BNCT was initiated in December 2023, with mean tumor dose of 50.22 GyE, which was combined with targeted antiangiogenic bevacizumab therapy. Follow-up brain MRI in February 2024 showed regression of the recurrent tumor (Fig. 1E).

**Figure 3.**
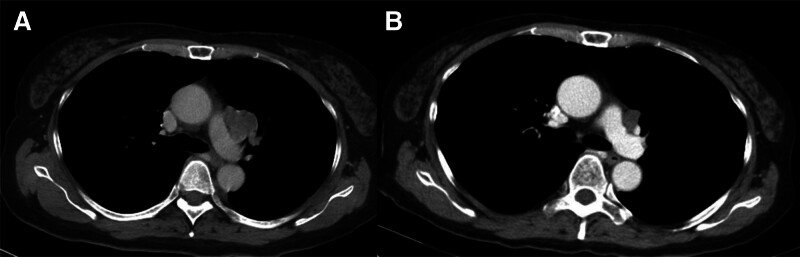
Pulmonary metastases. (A) Computed tomography (CT) showing metastatic lesions in the left pulmonary trunk. (B) Follow-up chest CT showing tumor regression 4 months after administration of systemic chemotherapy.

However, the patient presented with cough and hoarseness in March 2024, and chest X-ray revealed a left hilar mass, while chest CT revealed a poorly contrast-enhanced lesion in the region of the left pulmonary trunk, suggesting a newly-growing lung tumor or metastasis from the brain meningioma (Fig. 3A). Thoracoscopic exploration was attempted to excise the lung tumor but only an incisional biopsy was performed as the tumor was encased in the pulmonary artery. Histopathological examination showed spindle and epithelioid tumor cells with obvious nuclear pleomorphisms and numerous mitoses, consistent with lung metastasis from intracranial malignant meningioma (Fig. 2B). A drug regimen including the immune checkpoint inhibitor (ICI) pembrolizumab and antiangiogenic agent’s bevacizumab combined with the conventional chemotherapy agent’s 5-fluorouracil and paclitaxel was started in April 2024. The symptoms of cough subsided and follow-up chest CT in September 2024 showed tumor regression (Fig. 3B). Subsequent follow-up MRI also revealed a relatively stable brain condition (Fig. 1F) and the patient remains alive as of November 2024 at the time of writing this report.

## 3. Discussion

For all meningioma grades, extent of resection is reported to be the most important factor affecting outcome.^[4,14]^ However, maximal safe resection remains the primary therapeutic goal, as achieving complete resection is often challenging due to the infiltrative nature of nervous and vascular malignant tumor structures. Phase II, population-based, and retrospective studies have reported that radiotherapy can improve local control of nonbenign meningiomas,^[4,15]^ but our previous population-based retrospective analysis found no survival benefit of adjuvant radiotherapy for (grade III) malignant meningioma.^[3]^ In the current case, the patient exhibited a poor response to adjuvant radiotherapy, with evident meningioma recurrence occurring just 4 months after treatment.

Treatment of local recurrence at the primary tumor bed poses additional challenges. Currently, the major treatment modalities for recurrent meningioma are symptomatic control, salvage surgery, and radiotherapy. Salvage craniotomy is often the preferred treatment option but complete resection may be impossible for deep-seated or infiltrating tumors.^[6]^ Moreover, tumor recurrence within brain regions previously treated with high-dose radiotherapy may preclude re-irradiation due to the constraint of the maximum tolerable brain dose. In the current case, BNCT as compassionate targeted salvage treatment was advised on ground of the higher uptake of boron-containing drugs by malignant tumor cells relative to normal brain tissues allows for the delivery of a higher therapeutic dose to previous treated recurrent meningioma.^[13]^ Takai et al reported a median progression-free survival of 13.7 months and median overall survival of 29.6 months among 44 patients with recurrent or refractory high-grade meningioma receiving salvage BNCT in Japan.^[11]^ Similarly, Lan et al reported that a mean tumor dose of 29.4 GyE via BNCT resulted in 2 cases of stable disease, 1 partial response, and 1 complete response among 4 cases of recurrent intracranial meningioma in Taiwan.^[8]^ In our case, the patient received 2 rounds of BNCT administered by the same group in Taiwan, with mean tumor doses of 41.52 and 50.22 GyE, and achieved significant tumor regression and symptom mitigation after targeted radiation. While the salvage response of BNCT is promising, further research is needed to substantiate the long-term efficacy for local control and prolonged survival of recurrent high-grade meningioma.

This report also identified extracranial lung metastasis after repeated recurrence. Meningiomas are typically benign, but malignant meningiomas have a higher rate of recurrence and can progress to extracranial metastasis. However, distant metastasis is exceedingly rare, with an overall reported incidence between 0.1% and 0.2%. The lung is the most common site of metastasis, accounting for 35% of documented cases, followed by bone, spinal canal, and liver.^[16]^ Most metastatic meningiomas ultimately feature late pulmonary metastases. A literature review identified 45 published cases of WHO grade I to III meningioma progressing to single or multiple pulmonary metastases reported from 1995 to 2014. Further, almost all recurred after curative surgery.^[17]^ Another review identified 54 cases of intracranial meningiomas progressing to extracranial metastasis, of which 80% were pulmonary metastases occurring 2 months to 26 years after the primary tumor.^[18]^ The current case progressed to pulmonary metastasis around 23 months after the initial craniotomy. Notably, the primary tumor is often the source of metastasis, suggesting that the primary tumor is inadequately controlled. The clinical and pathological features that predispose meningiomas to metastasis remain poorly understood due to the limited number of documented cases.

There is currently no widely accepted treatment for pulmonary or systemic metastases from intracranial meningioma. Recommended therapies include various drug regimens consisting of small molecule protein kinase inhibitors, antiangiogenic drugs, ICIs, and peptide receptor radionuclides.^[19]^ In the current case, we prescribed the ICI pembrolizumab and the antiangiogenic drug bevacizumab based on a phase II study reporting that pembrolizumab alleviated recurrent and residual high-grade meningioma, with a median progression-free survival of 7.6 months and median overall survival of 20.2 months.^[19]^ The partial response achieved using pembrolizumab, bevacizumab, and chemotherapy supports further investigations on the efficacy of this regimen.

## Acknowledgments

The authors would like to thank Enago® for the English language review.

## Author contributions

**Conceptualization:** Sheng-Yow Ho.

**Data curation:** Li-Ching Lin, Ya-Ju Wu, Yuan-Hung Wu, Wan-Lin Shen, Chin-Hong Chang, Sheng-Yow Ho.

**Supervision:** Sheng-Yow Ho.

**Writing – original draft:** Wan-Hsuan Chow, Sheng-Yow Ho.

**Writing – review & editing:** Sheng-Yow Ho.
